# Micro-particle entrainment from density mismatched liquid carrier system

**DOI:** 10.1038/s41598-022-14162-5

**Published:** 2022-06-13

**Authors:** S. M. Naser Shovon, Adeeb Alam, William Gramlich, Bashir Khoda

**Affiliations:** 1 Department of Mechanical Engineering, The University of Maine, Orono, ME USA; 2 Department of Chemistry, The University of Maine, Orono, ME USA

**Keywords:** Mechanical engineering, Particle physics

## Abstract

Micro-scale inorganic particles (d > 1 µm) have reduced surface area and higher density, making them negatively buoyant in most dip-coating mixtures. Their controlled delivery in hard-to-reach places through entrainment is possible but challenging due to the density mismatch between them and the liquid matrix called liquid carrier system (LCS). In this work, the particle transfer mechanism from the complex density mismatching mixture was investigated. The LCS solution was prepared and optimized using a polymer binder and an evaporating solvent. The inorganic particles were dispersed in the LCS by stirring at the just suspending speed to maintain the pseudo suspension characteristics for the heterogeneous mixture. The effect of solid loading and the binder volume fraction on solid transfer has been reported at room temperature. Two coating regimes are observed (i) heterogeneous coating where particle clusters are formed at a low capillary number and (ii) effective viscous regime, where full coverage can be observed on the substrate. ‘Zero’ particle entrainment was not observed even at a low capillary number of the mixture, which can be attributed to the presence of the binder and hydrodynamic flow of the particles due to the stirring of the mixture. The critical film thickness for particle entrainment is ${h}^{*}=0.16a$ for 6.5% binder and ${h}^{*}=0.26a$ for 10.5% binder, which are smaller than previously reported in literature. Furthermore, the transferred particle matrices closely follow the analytical expression (modified LLD) of density matching suspension which demonstrate that the density mismatch effect can be neutralized with the stirring energy. The findings of this research will help to understand this high-volume solid transfer technique and develop novel manufacturing processes.

## Introduction

Improved understanding of higher yield particle transfer processes during dip coating is critical to address current challenges facing the manufacture of next-generation materials and devices, including tubular structures, synthetic blood vessels^1^, tissue scaffolds^2^, flexible electronics^3^, filtration systems^4^, and meta-surfaces that regulate optical, acoustic, and magnetic waves^5^. Often, to achieve these functions, the solid substrate (i.e., flat plate, cylinder, convex surface^6^) is dipped into a liquid bath of colloids^7^, sol-gels^8,9^, or suspensions^4,10^, which contains the positively or neutrally buoyant solid particles (usually sub-micron size) that will be delivered spatially. Particle transfer occurs at the three-phase boundary^11^ during the withdrawal stage due to entrainment, when the viscous drag force becomes larger than the resistive capillary force. The simplicity, low cost, and reasonable control make this particle transfer process by dipping an extensively used method^12,13^.

Delivering or depositing nano-materials (*d* < 30 nm) on a substrate by dipping into homogeneous density-matching solutions are commonly investigated for surface passivation^14^, selective activation of surface-energy^15^, thermal barrier, antifouling, surface filtration^16^ and meta-surface wave absorption or manipulation^17^. To facilitate the delivery of small particles, they are often entrained into a solution called a liquid carrier system (LCS). Both aqueous^18^ and organic solvent carriers are commonly used as LCS, where a lower volume fraction of monodisperse particles (< 20%) forms a suspension. Leveraging the rheological properties of the mixture, particles are delivered on the substrate, including internal surfaces and porous architectures with possibly low volumetric flux. Dip coating can also deliver particles in complex (e.g., bifurcated or branched) geometries such as refractory material coat for foundry cavity^19^ and bio-compatible polymer coat to construct synthetic blood vessels^1^. Aditionally, dip coating is often used for tissue scaffolds fabrication^2^, porous structure joining^20,21^, oil/water separation^22^, surface protection^23–25^, soft robotics locomotion^26^ and flexible electronics isolation^3^.

A method to predict the dip coated film thickness on a plate geometry withdrawn from a non-evaporating, Newtonian fluid was first proposed in 1942 by Landau and Levich^27^ and then Derjaguin, using the LLD equation, $$h=0.94{l}_{c}{Ca}^{2/3}$$. The liquid film thickness $$(h)$$ depends on both fluid properties (i.e., surface tension $$\gamma $$, density $$\rho $$, viscosity $${\eta }_{0}$$) and dipping process parameters (i.e., withdrawal velocity $$U)$$. These parameters are incorporated in the LLD equation with the dimensionless capillary number, $$Ca=\frac{{\eta }_{0}U}{\gamma }$$ and capillary length, $${l}_{c}=\sqrt{\gamma/\rho g}$$ for $$Ca<{10}^{-2}$$. It was later expanded for cylindrical geometries, since wires, ropes, fibers or tubes are extensively used in industrial, medical, and textile applications^29–31^. To define the cylindrical geometry, the Goucher Number, $$Go= r/{l}_{c}$$, is often used which is the ratio between the vertical curvature expressed by capillary length, $${l}_{c}$$ and the azimuthal curvature expressed by fiber radius,$$r$$. The modified LLD theory for cylindrical geometry is applicable for $$Go<3$$ and is expressed as $$h=1.34r{Ca}^{2/3}$$ to predict the liquid film thickness on wires and fibers^28,29^.

When particles are added to the LCS, it starts to demonstrate complex behavior, which has received substantial attention in the literature. Solid particles in a liquid are considered obstacles to its rheological behavior and increase viscosity. At a relatively high particle volume fraction, the viscosity of the particle-laden mixture starts to follow a non-linear behavior. This non-Newtonian viscosity is commonly described by apparent viscosity $({\eta }_{r}=\tau/\dot{\gamma })$ where $\tau$ is the shear stress and $\dot{\gamma }$ is the shear rate. The apparent viscosity for a suspension can be normalized and reported as ${\eta }_{r}=\frac{\eta (\phi ) }{{\eta }_{0}}$. Einstein first described a formula for the viscosity of suspension with low particle concentration^30,31^. This formula was then adjusted by Krieger and Dougherty^32^ for high solid concentration, which provides effective viscosity for low to high shear rates^32–34^. Several other studies have described the suspension rheology^35^, clogging in confined flows^36,37^ or the inertial suspension flow^38^ for wet deposition of sub-micron particles.

Larger inorganic micro-scale particles ($d >1$ µm) are commonly used in manufacturing industries (e.g., brazing powder, metal filler, and 3D printing powder). Understanding their transfer process from liquid carrier system can be beneficial for micro-nano manufacturing processes. For example, in transient liquid phase bonding, the interlayer (filler) metal particles need to be delivered on the faying surface for joining. By understanding their transfer physics from multi-phase fluid can open-up an economical manufacturing process for complex architecture including porous solid^20,21^. Such understanding can be directly implemented to novel particle sorting technique of different size and density distribution^39,40^, inorganic material coating on 3D printed polymer surface^41^, acoustic meta-surface for hypersonic boundary layer transition and stabilization^42^, dust mitigation in both hydrophobic and hydrophilic surfaces^43^ surface wettability and flowability improvement for medical devices^44^ and thin-surface wicks for heat and mass transfer^45–47^. The micro-scale inorganic particles have reduced surface area and higher density, making them negatively buoyant in the LCS and challenging to yield high solid transfer due to the density mismatch causing them to settle in suspension. Additionally, due to the economical droplet-based fabrication techniques used (e.g., gas atomization, plasma atomization, and plasma rotating electrode process), the particle size distribution often follows a continuous, polydisperse exponential pattern, which is commonly expressed with the Rosin–Rammler expression^48^. Their high yield delivery to hard-to-reach places (i.e., porous structure, inner surfaces) and large surface areas is often a functional requirement. The transfer mechanism of polydisperse particles is rarely investigated in this dip-coating process with a density mismatched heterogeneous mixture.

Earlier particle transfer by dip-coating research efforts can be classified broadly: (i) adhesion and peel test measurements of film strength, (ii) mono-layer formation of nano-particles, (iii) effect of process parameters (i.e., dipping angle, withdrawal speed, dwelling time, etc.) on the deposited layer, and (iv) characterizing the interfacial interactions at the molecular level^49^. The particle–surface adhesion force for entrainment can be categorized as: (i) electrostatic, (ii) van der Waals, (iii) covalent bond (iv) hydrogen bond and (iii) gravitational forces^50–52^. For submicron size particles, the electrostatic force and van der Waals are prominent for adhesion to the substrate due to their large specific surface area. However, for larger particles ($>1$ µm), the specific surface area is reduced, which makes them non-interacting and non-agglomerating particles in the liquid matrix or otherwise known as the non-Brownian regime^20,53,54^. The mechanism of non-Brownian microparticle (avg. Dia 40 ~ 550 µm) transfer has been studied by Palma et al.^10^ and *Gans* et al.^4^ using particles with a spherical morphology. The entrainment of the neutrally buoyant micro-particles in these studies was found to be directly related to the dimensionless capillary number. However, due to the higher particle to liquid density ratio, inorganic microparticles are prone to sedimentation in most liquid carrier systems and require additional considerations. As a result, additional kinetic energy is required to ensure the uniform dispersion of the particles in a ‘pseudo suspension’. Due to this additional complexity, particle transfer of large (> 1 µm) inorganic, density mismatched, polydisperse particles from a stirred mixture with high solid loading ($>20$%) has not been fully investigated in the existing literature.

In this work, we investigate the transfer of micrometer-size inorganic powder particles from density mismatching mixture through the dip-coating process on a cylindrical substrate. A liquid carrier system (LCS) solution was prepared by combining a polymer binder and an evaporating solvent. Nickel-based brazing powder with a spherical shape and high density were used as the solid particles added to the LCS solution to prepare the density mismatching mixture. Particles were dispersed with the assistance of a magnetic stirrer to maintain the ‘pseudo suspension’ characteristics of the mixture. The particle transfer mechanism was characterized by surface coverage, film thickness, particle layer, solid loading, and the binder volume fraction. Based on the observations, the particle transfer process is differentiated into two regimes: (i) heterogeneous regime and (ii) effective viscous regime, which is parametrized with the dimensionless capillary number. The transferred particle matrices are compared with the analytical expression of a density matching suspension (modified LLD equation). We found that in the presence of binder, particles can be entrained on the substrate even below the critical film thickness (${h}^{*}\ge 1.1a,$ where *a* is the particle radius) reported earlier^55^. Furthermore, the transferred particle matrices of our ‘pseudo suspension’ is compared with the existing analytical expression of density matching neutrally buoyant suspension.

## Methodology

**Figure 1:**
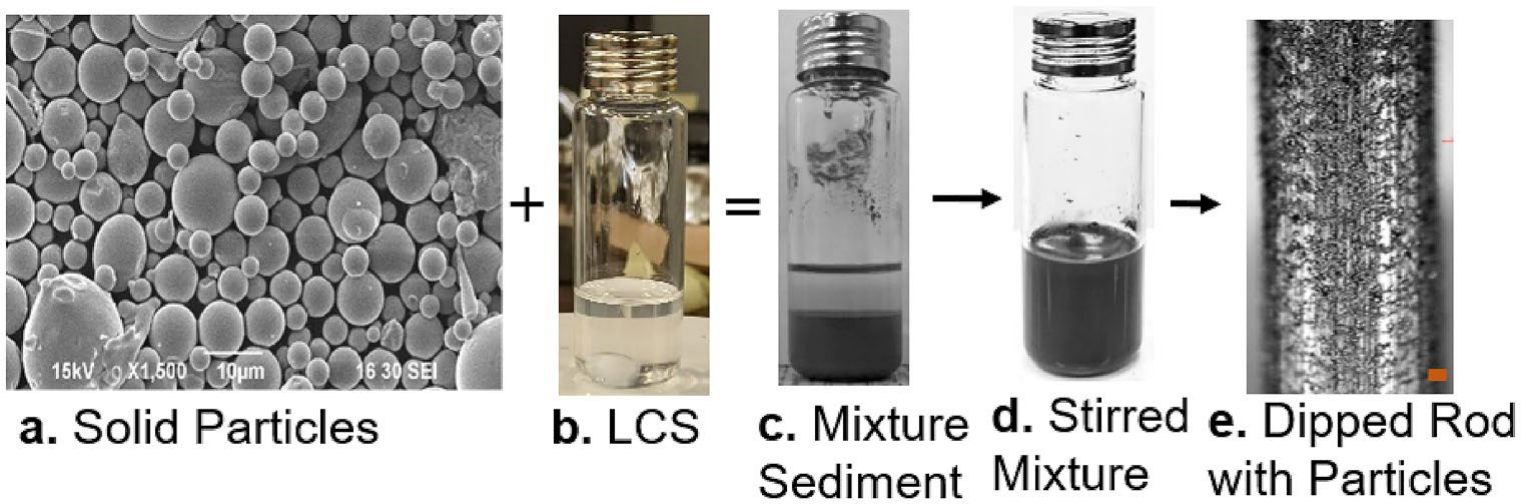
SEM micrographs of particles added to the homogeneous LCS to make the heterogeneous mixture. The mixture is stirred, and the rod is dipped for particle transfer.

In this study, the density mismatching mixture was prepared with three components: binder, solvent, and solid particles. The binder and solvent acted as a liquid carrier solution (LCS) which helped transfer solid particles on the substrate. The polymethyl methacrylate (PMMA; M_W_ ~ 15,000 g/mol); Sigma Aldrich) was the binder, which has low density (~ 1.19 g/cm^3^), ~ 41 mN/m surface tension, and is a benign, non-explosive and non-flammable material^56^. 1,3-Dioxolane (from Sigma Aldrich) was used as the solvent, which has 34.3 mN/m surface tension, ~ 1.06 g/cm^3^ density, and vapor pressure of 79 mmHg at 20 °C^57^. Brazing powder (Nicrobraz 51; Mesh 325; Wall Colomonoy Company, Ohio) was used as the solid inorganic particle (IP), which has a density of 7.89 g/cm^3^. Before mixing with LCS mixture, the particles were sieved with a Gilson Performer III shaker through a stainless steel 635 mesh to reduce its average particle size. After sieving, the average particle diameter was measured by analyzing the SEM image in ImageJ, which showed a 5.72 µm average diameter and a standard deviation of 3.09. The dipping mixture was prepared in 20 mL screw top, clear borosilicate glass, vial with the dimension of $75.5 \times 22.5$ mm provided by Fisher Scientific. The mixture preparation is shown in Fig. 1 and discussed in detail elsewhere^54^.

The density mismatch between particles (7.89 gm/cm^3^) and the LCS solution will facilitate fast sedimentation of particles and drag them to the bottom. To resist the gravitational force, external kinetic energy in the form of agitation was applied to maintain a just suspending speed for the particles. A cylindrical magnetic stirrer was used that had a length and diameter of 14.9 mm and 5.95 mm, respectively. Stirring created a pressure difference (normal stresses so that the particles lifted off and stayed suspended, creating a dispersed mixture (‘pseudo suspension’). Cylindrical AISI 1006 mild steel rod with an average diameter of 1.06 mm (procured from ClampTite LLC) was used as the substrate for transferring particles. Rod samples were cleaned in an ultrasound bath with acetone for 10 min at 50 ºC to remove any surface contaminant and passive film. The effective shear viscosity of the polymer solution (LCS) was determined by a rotating rheometer (Anton Paar Moduler Contact Rheometer; MCR302) using a flow curve test with shear rates ranging from 2 to 1000 s^−1^ and using a parallel plate geometry with 50 mm diameter and a gap of 0.2 mm. The surface tension and density of the LCS solution was calculated by the weighted average of binder and solvent^10,58^, which can be expressed as $\gamma ={\gamma }_{sv}+(1-{\phi }_{s}){\gamma }_{b}$ and ${\rho }_{s}={\rho }_{sv}+(1-{\phi }_{s}){\rho }_{b}$ Where $\gamma , \phi ,$ and ${\rho }_{s}$ denote for surface tension, volume fraction, and density of LCS solution, ${\phi }_{s}$ for solvent volume fraction, and the subscript $sv {\text{and}} b$ denotes solvent and binder, respectively.

**Figure 2:**
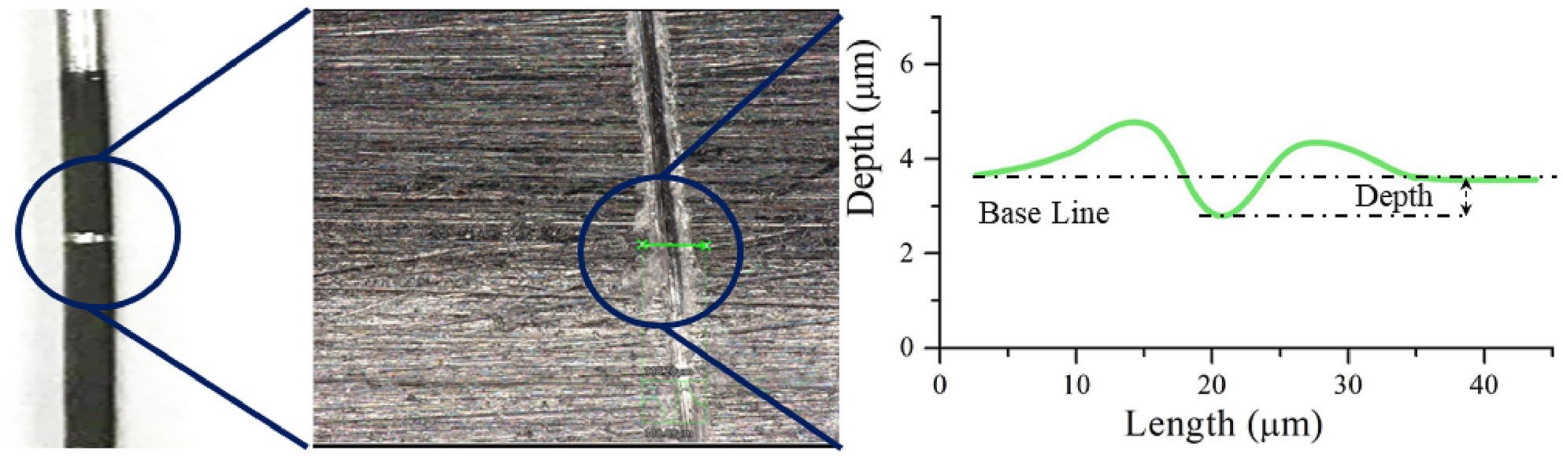
Sample image of the 3D depth profile of liquid coating.

An in-house dipping station was used in our lab for this work. The construction of the dipping setup and the working process were discussed in our earlier work^48^. The dipping experiment was performed at 25 °C and atmospheric pressure with a low relative humidity of 20%, which allowed evaporation of solvent from the liquid–vapor interface (meniscus) during the dipping process. The substrates were immersed into the density mismatching mixture at a constant speed (10 mm/s) and maintained for 10 s of immersion time, followed by the withdrawal at the same hoisting speed. The samples were kept at room temperature between 2–4 h at a vertical position for drying before the polymer film thickness was measured using a VHX 7000 Digital 4 K microscope (KEYENCE corp., IL). A narrow groove was created with a round edge cut on the substrate at three different locations and the 3D profile depth was measured and averaged as shown in Fig. 2. High resolution 4 K images of the dipped rod were taken with the same microscope and the images were analyzed with a script written in MATLAB. The image was transformed into gray scale and binary segmentation was performed using Otsu method^59^. Particles were separated by tracking the center using watershed algorithm and the particle boundary is identified with the closed contour. The length scale was measured for their average size. Additionally, the surface coverage was measured with the ratio of the area covered by the particle to the total area of the substrate surface at three locations (bottom, middle and top) using the same image analysis script.

## Results

### Solid loading of dipping mixture

Adding binder and immiscible particles to a liquid transforms its rheological properties. The addition of particles will act as obstacles to the fluid flow which will induce a non-linear dependency under applied shear (non-Newtonian). At a higher volume fraction, the mixture may exhibit friction-induced continuous shear thickening (CST) or discontinuous shear thickening (DST)^60^, causing a stress-induced solid-like shear jammed state. Similarly, adding binder will increase the viscosity, which may facilitate the particle adherence, but it may cause non-uniform coating due to the high viscosity nature of the mixture. The jamming volume fraction for random close packing (RCP) density is about 64%, which may increase to ~ 74% for polydisperse particles^61^. In this paper, 5.69 µm average diameter particles with volume fraction (${\phi }_{p})$ 20%, 35%, and 50% were investigated with 2.5%, 6.5% and 10.5% binder volume fraction (${\phi }_{b})$. The remaining mixture contains solvent which can be defined as ${{\phi }_{s}=1-\phi }_{p}-{\phi }_{b}$ as shown in Table 1.

**Table 1:** Composition of binder, powder, and solvent in the heterogeneous mixture.

Mixture composition	% Volume Fraction		
	Binder (${\phi }_{b})$	Powder (${\phi }_{p})$	Solvent ${(\phi }_{s})$
00	2.5	20	77.5
01	2.5	35	62.5
02	2.5	50	47.5
10	6.5	20	73.5
11	6.5	35	58.5
12	6.5	50	43.5
20	10.5	20	69.5
21	10.5	35	54.5
22	10.5	50	39.5

### Characterizing the liquid carrier system

**Figure 3:**
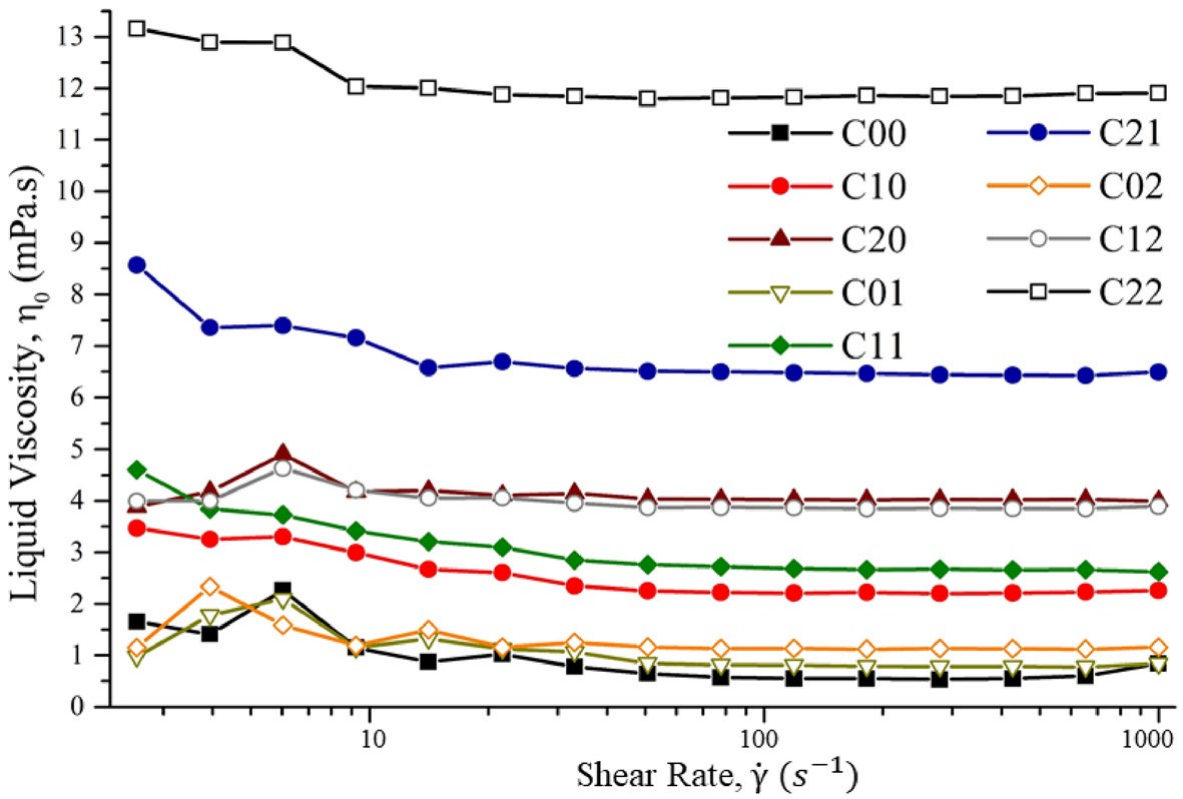
Viscosity versus shear rate diagram for the liquid solution. The C00 to C22 corresponds to the compositions in Table 1, respectively.

The viscosity versus shear rate data for the LCS was measured by the rheometer and is plotted in Fig. 3. Little variation in viscosity (${\eta }_{0})$ is observed at different shear rates, presenting the Newtonian behavior of the LCS solution. The viscosity of the 1,3-Dioxolane solvent was reported as 0.58 mPa s at room temperature^62^, which demonstrate that adding PMMA increases the viscosity of the LCS solution. It should be noted that the viscosity of the most dilute LCS (C00) solution was found slightly lower than the solvent viscosity at higher share rate using 50 mm parallel-plate geometry. This can be attributed to the very low viscosity and resulting torque which are close to the limits of the instrument. Concentric cylinders or a cup attachment geometry may provide more accurate viscosity for very dilute solution, however the parallel plate geometry was used because of the range of viscosities tested. Expected film thickness after dipping is determined by Eq. (1) which combines the capillary number and Goucher number^55^ expressed as

1 $$h=\frac{1.34r{Ca}^{2/3}}{1+2.53{{G}_{o}}^{1.85}/[1+1.79{{G}_{o}}^{0.85}]}$$

**Figure 4:**
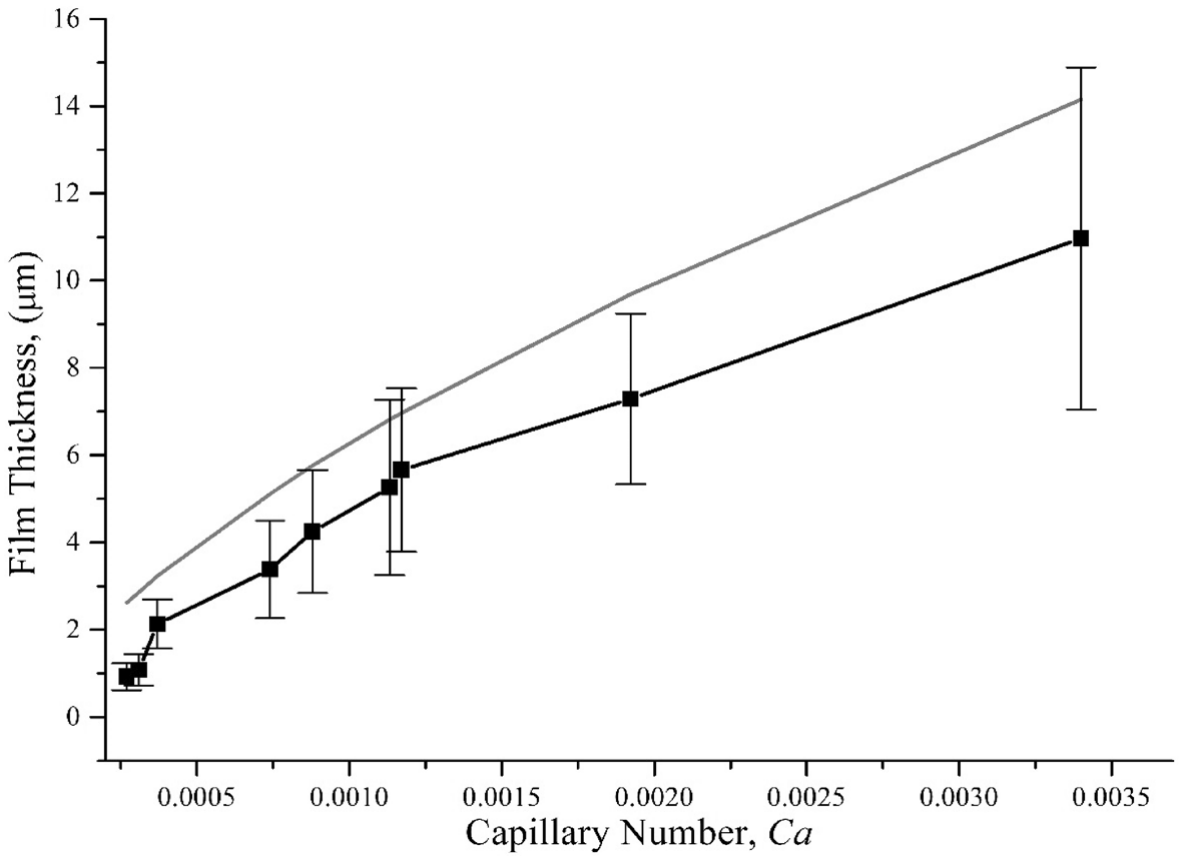
Polymer film thickness as a function of capillary number. The grey line corresponds to the expected (theoretical) liquid thickness. The solid rectangular points represent the experimentally measured film thickness with standard error shown for each data point.

Both the experimental and expected film thickness are shown in Fig. 4. The capillary number relied upon the viscosity variation due to binder volume fraction while keeping the withdrawal speed at 10 mm/s (medium speed). However, the experimental measurement always lags the semi-empirical equation (Eq. 1) by Dincau et al.^55^, which is derived for a non-evaporating liquid and thus, considered a wet film thickness. In evaporating solvent case, the resultant film thickness is governed by the evaporation rate, which is impacted by the diffusivity of the polymer–solvent system. As the solvent evaporates, a dense polymer rich skin layer forms at the liquid vapor interface decreasing the diffusivity and increasing the resistance to solvent evaporation^63^.

**Figure 5:**
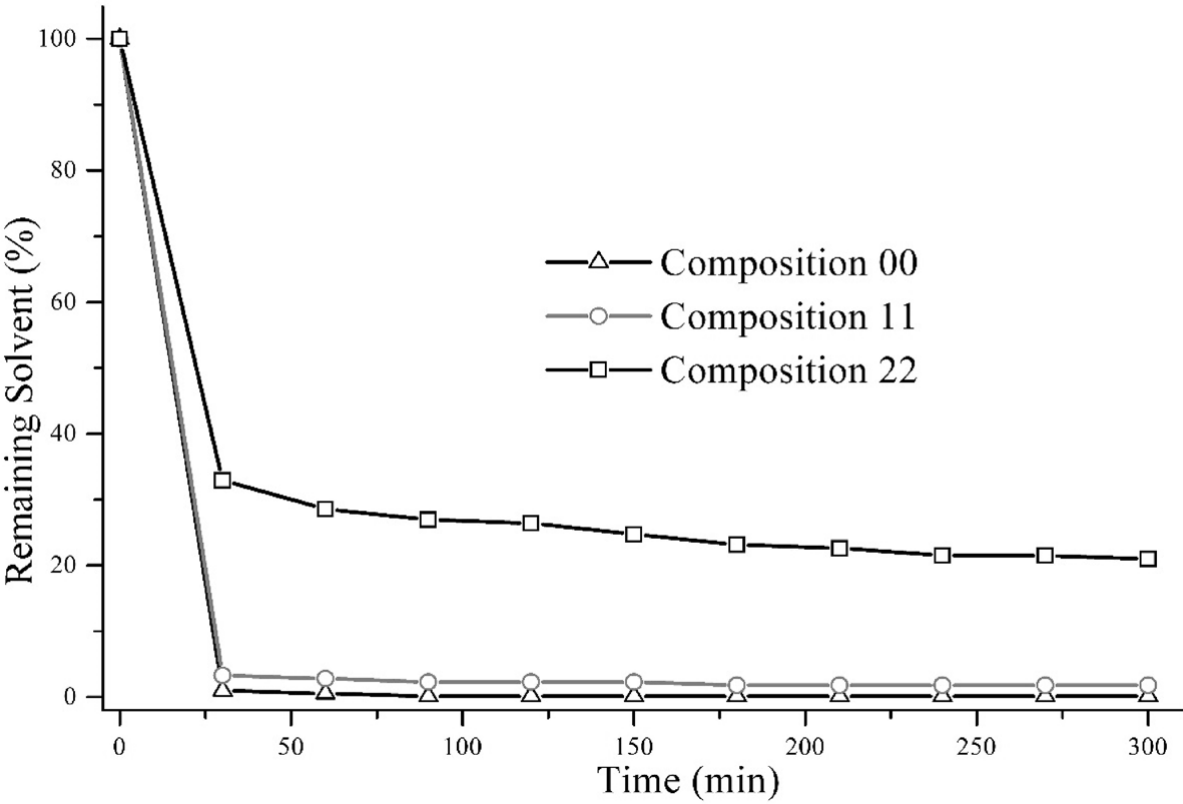
Solvent evaporation from the LCS in terms of remaining solvent amount (%) vs time (min).

To understand the evaporation rate and the polymer film compactness of our LCS system, a droplet test was performed. Three LCS compositions (00, 11 and 22) with 3.5, 11.1 and 23 wt. % binder were investigated for solvent evaporation. A 23 µL droplet of LCS solution was placed on a glass slide at the same conditions as the dipping experiment (20 °C) and the mass was measured every 30 min for a total of 300 min. The difference between the mass is due to the evaporation of the solvent which is plotted in Fig. 5. The evaporation rate is high during the initial 30 min and it evaporates little afterwards. Additionally, more solvent evaporates at the lower binder concentration than the higher binder concentration. For example, 67.1%, 96.7% and 99% of total solvent weight evaporated from composition 22, 11 and 00 after 30 min, respectively. This phenomenon contributes to the difference between expected and measured film thickness and describes the lower thickness in the measured film shown in Fig. 5. As the evaporation continues at a different rate for different compositions after dipping, the generated film will have a hard surface and viscous core, potentially generating a porous film. As a result, the coated film is relatively easy to remove which has been also observed in our experiment.

### Characterizing the dipping mixture

The addition of particles into a LCS can form an emulsion^64^ or a suspension^65,66^, or a solidus mixture^61^, which often demonstrate non-linear rheological behavior (i.e., shear-thinning or thickening) under an applied force. Particle laden suspensions have been divided into three different regimes: dilute (${\phi }_{p}\le 0.1),$ semi-dilute (${0.1<\phi }_{p}\le 0.25$), and concentrated (${\phi }_{p}>0.25$)^67–69^. In dilute or semi-dilute regimes, the viscosity mostly maintains linear rheological behavior (like Newtonian), which can be often measured experimentally with a rheometer^61^. However, in the presence of a high-volume fraction, inorganic micro-particle, measuring the viscosity with a rheometer is challenging as it gives inconsistent results due to the plate-particle contact. The Krieger & Dougherty model^32^ has been used in the literature^70,71^ to predict the mixture viscosity, which correlates the LCS viscosity with the volume fraction of particles (Eq. 2).

2 $$\eta \left({\phi }_{p}\right)={\eta }_{0}{\left(1-\frac{{\phi }_{p}}{{\phi }_{p,max}}\right)}^{-B{\phi }_{p, max}}$$

In Eq. (2), $${\eta }_{0}$$ is the liquid viscosity, $${\phi }_{p}$$ is the particle volume fraction, $${\phi }_{p, max}$$ is the maximum packing of the powder and $$B$$ is the Einstein coefficient, which is dependent on the particle shape. For random close packing of spherical particles, $${\phi }_{p, max}=0.64$$ ^72^ and $$B=2.5$$. The dimensionless capillary number ($$Ca)$$ of the mixture is the determined by using the mixture viscosity as: $$Ca\left({\phi }_{p}\right)=\frac{U}{\gamma }\eta \left({\phi }_{p}\right)$$.

To disperse the high-density particles in the LCS, the mixture was stirred to maintain the just suspended speed while minimizing the vortex. Measuring the impact of the Brownian motion and inertia effects is necessary because they induce additional bulk stress in a suspension^73^. Both the Peclet number^68,69^, $Pe=\frac{6\pi {\mu }_{0}{a}^{3}\dot{\gamma }}{kT}$ and the Reynolds number^70^, $Re=\frac{{\rho }_{0}{a}^{2}\dot{\gamma }}{{\mu }_{0}}$ were determined for the mixture at shear rate 2 and 1000 s^−1^. Here $k=1.38\times {10}^{-23}$ JK^−1^ is the Boltzmann constant, T is the absolute temperature (25℃), ${\eta }_{0}$ is the solution viscosity and $a$ is the average particle radius. The Peclet number ranges between 10^3^ to 10^6^ and Reynolds number ranges between 10^−9^ to 10^−12^ for our mixture, which falls within the Non-Brownian regime.

### Characterizing the particle transfer and coating

**Figure 6:**
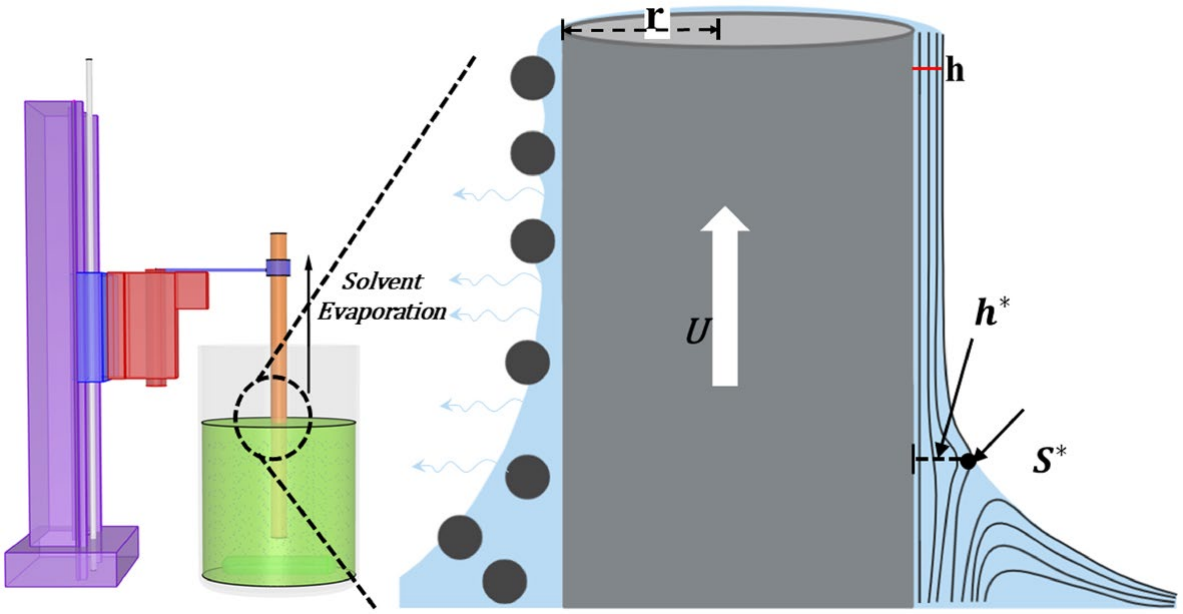
Schematic of fluid streamlines and dip coating mechanism. ${S}^{*}$ denotes for the stagnation point, $h$ for expected liquid thickness, ${h}^{*}$ for the critical film thickness, $r$ for fiber radius, and $U$ for withdrawal velocity.

**Figure 7:**
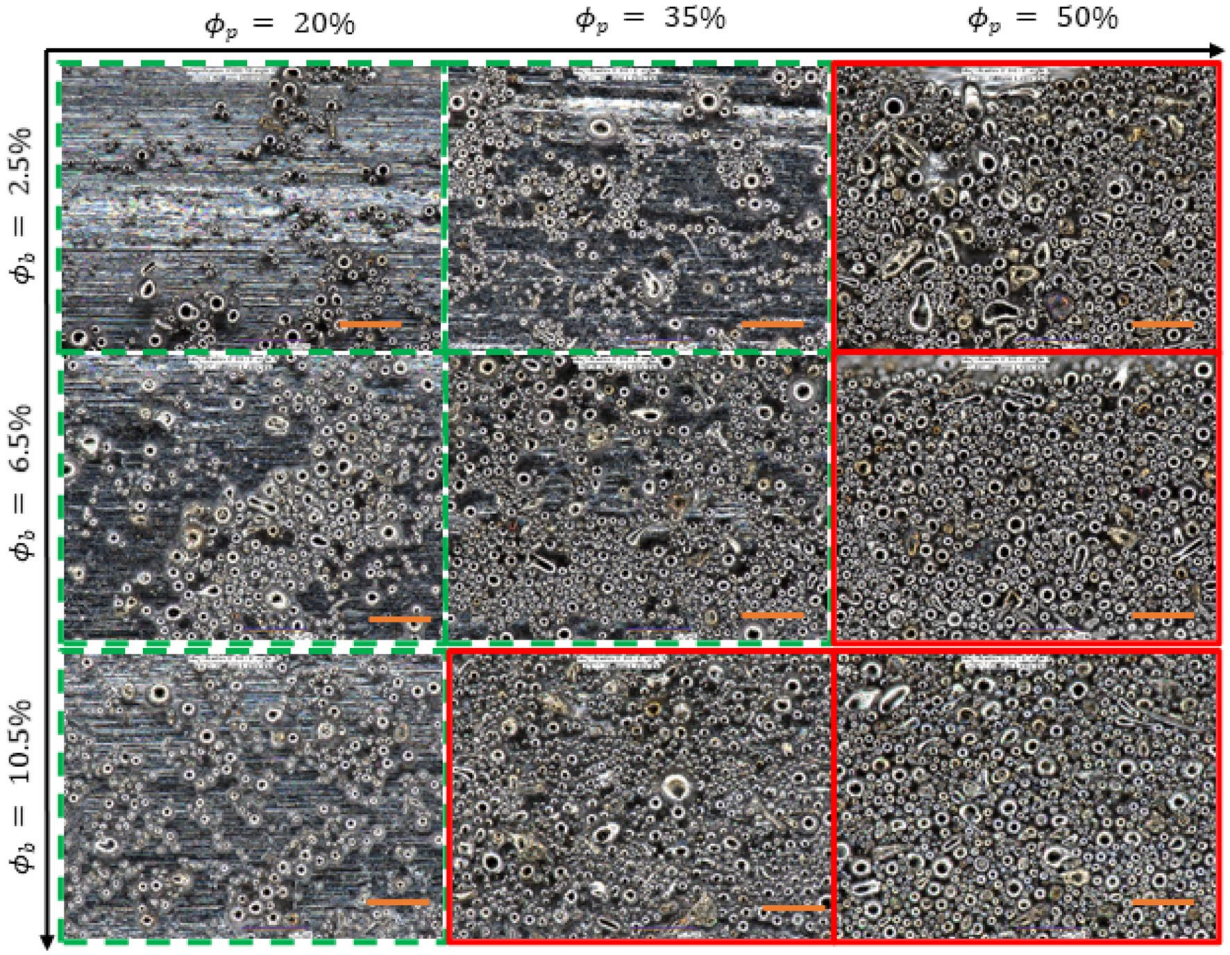
Surface morphology of the coated substrate at different volume fractions of binder (${\phi }_{b}$=2.5%,6.5% and 10.5%) and particles (${\phi }_{p}$=20%,35% and 50%). The pictures were taken 1000X zoom. The dashed green line bounded figures correspond to the heterogeneous regime and the red solid line bounded figures show the effective viscosity regine. 50 µm scale bar.

**Figure 8:**
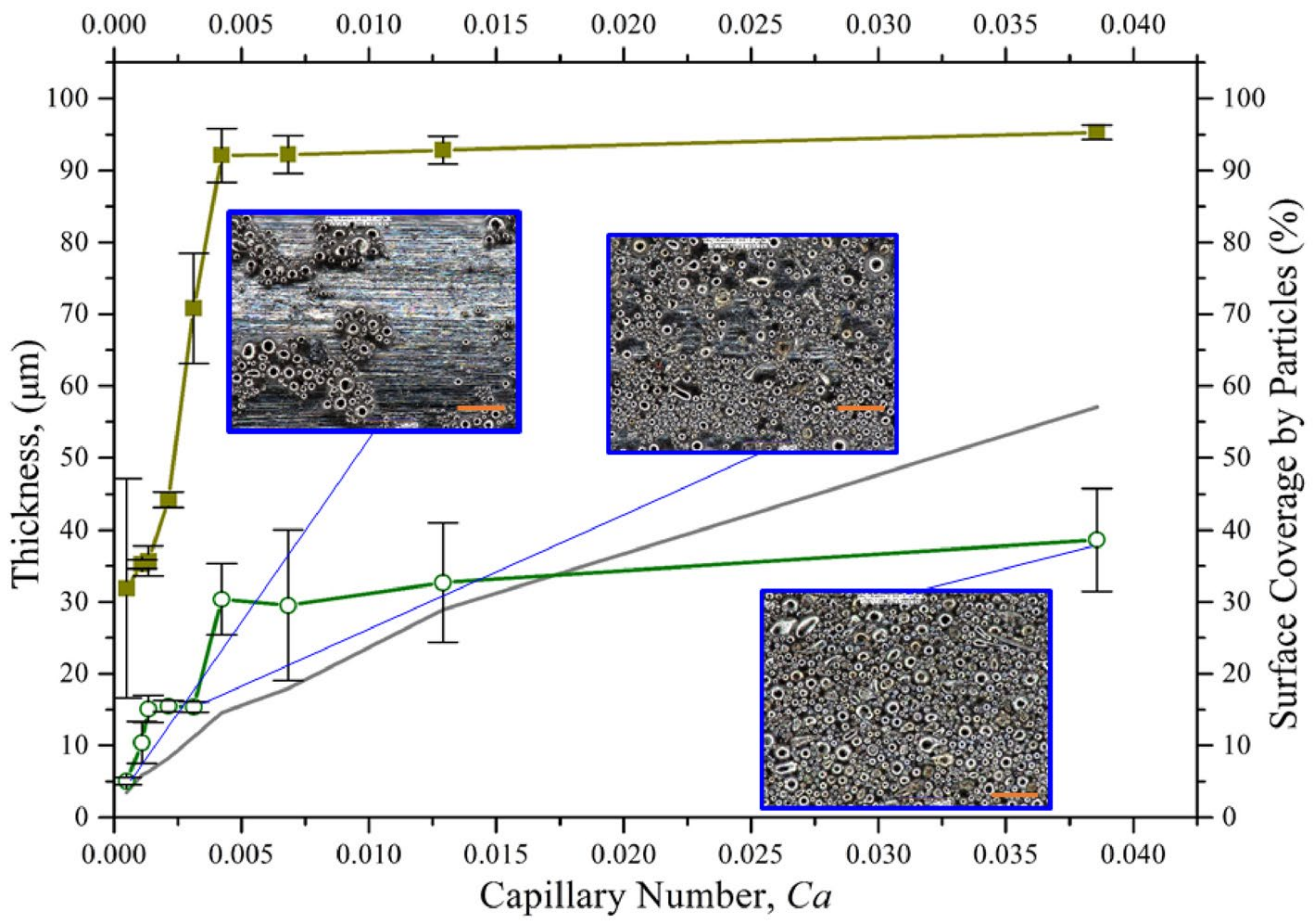
Surface coverage (%) and particle laden film thickness as a function of capillary number. The circular hollow dotted point represents the film thickness, and the rectangular solid dotted point represents the surface coverage by particles. 50 µm scale bar.

During the retraction of the substrate rod, the mixture velocity is directed downward, and the intermolecular forces between mixture and substrate surface help the particles adhere to the substrate. The solvent evaporation continues after extraction of the substrate and a dense polymer rich skin layer forms as shown in Fig. 6. The adhesive force exhibits enough attracting force to the solid particles to avoid the gravitational effects and helps the particles adhere to the substrate. Microscopic images of the coated rod at three different locations were used for each specimen to average the coating thickness and particle coverage as shown in Fig. 7. By changing the volume fraction of the mixture, two coating regimes are observed: (i) heterogeneous regime and (ii) effective viscosity regime. With a low particle volume fraction (${\phi }_{p}=20{\%}$), a coating film was created with fewer inorganic particles clustered into an irregular pattern as shown in Fig. 7. This coating regime is referred to as the heterogeneous regime because of the irregular formation. When the volume fraction of particles increased to 35% and 50% in the mixture, the liquid film entraps abundant particles to the substrate from the meniscus. Moreover, the high binder concentration (${\phi }_{b}=10.5{\%}$) increases the viscosity and decreases the evaporation rate, which also influences the adhesion of particles. Thus, a more uniform arrangement of the particles can be observed on the substrate, which is expressed as the effective viscosity regime shown in Fig. 7. These regimes are coherent with the regimes reported by recent literature with neutrally buoyant particles^4,10^. However, no ‘zero-particle’ regime can be observed here even at a lower capillary number. By using the theoretical suspension viscosity and the capillary number obtained by Eq. (2), the theoretical coating thickness was calculated and compared with our experimental data. Surface coverage is also measured and plotted against the capillary number in Fig. 8.

## Discussion

### Formation of the heterogeneous film

The entrainment of particles on the liquid film begins when the viscous drag force becomes larger than the resistive capillary force. The liquid film thickness, particle size, and volume fraction influence the entrainment of particles. At a lower volume fraction of binder and particles, the capillary effect remains high. As a result, fewer small particles are entrained in the relatively thin liquid film. Colosqui et al.^74^ provided a threshold capillary number, ${Ca}^{*}$, for 2D plate geometry with respect to the Bond number, ${B}_{0}={(\frac{a}{{l}_{c}})}^{2}$. Particles will assemble or entrain inside the meniscus at ${Ca}^{*}>0.6{{B}_{0}}^{0.75}$. The above condition is further modified as ${Ca}^{*}>0.24{{B}_{0}}^{0.75}$ by Sauret et al.^40^, reducing the threshold capillary number and hence the critical film thickness for entrainment. However, for cylindrical geometries like fiber and wires, Dincau et al.^55^ derived the expression considering the particle and cylinder radius as:

3 $${Ca}^{\boldsymbol{*}}\cong 0.645{\left[\frac{\alpha a}{3r}\left(1+\frac{2.53{Go}^{1.85}}{1+1.79{Go}^{0.85}}\right)\right]}^{1.5}$$

Here, $\alpha$ represents the complex shape of the meniscus around the particle and has been considered as $\alpha =1.1$ to determine the threshold capillary number^55^. The threshold ${Ca}^{\boldsymbol{*}}$ is calculated from the above equation as $7.95\times {10}^{-5},$ which is smaller than the $Ca$ for the LCS used in this work, and therefore, indicating particle entrainment from the meniscus. However, fewer particles are observed in this regime and they are often clustered as shown in Fig. 6. The effects of convective flux acting on the particle-laden mixture varies due to particle polydispersity, which creates a variation in the viscous drag force for entrainment. We observed that the particle coated thickness is higher than the expected (theoretical) values, which is derived for homogeneous precursor with monodisperse particles. This increase in thickness of the particle laden film can be attributed to the entrainment of large particles ($>10 \mu m$) on the substrate increasing the average thickness above the expected value. Moreover, the continuous evaporation of solvent increases the capillary forces on the surface to cause the particles to cluster. Lower polymer content will enable quicker evaporation and a thinner film. As the polymer film dries, its capillary forces between the particles will pull the particles together to form the clusters, creating a heterogeneous regime which can be observed in Fig. 7.

### From heterogeneous to the effective viscous regime

In the effective viscous regime, particle entrainment is significantly higher, which can be seen in the coverage. Multi-layer particle entrainment is also observed in this regime. As the particle volume fraction and binder concentration increase, the rheology of suspension will demonstrate non-linear behavior (non- Newtonian), and the effective viscosity will change with the applied shear rate. Both surface tension and viscosity of the particle-laden mixture will increase, which will cause the capillary number to increase. Due to the complex viscous behavior, the LLD equation can’t accurately predict the coating layer thickness, which can be seen in Fig. 8. Different researchers have previously studied dense suspension. Bonnoit et al.^75^ studied 20 to 140 µm dia. neutrally buoyant particles at 15 to 55% volume fraction and they showed deviation from Newtonian viscosity at 40% volume fraction. Similar non-Newtonian behavior is also reported for spherical particles over 40% volume fraction which often demonstrate shear thinning behavior^70^. The particle morphology (size and shape) in the suspension defines its rheological behavior and at a higher volume fraction (> 50%), the particle-laden suspension may exhibit solid-like shear jammed state^76–78^. Dip coating has been performed with dense suspensions (${\phi }_{p}=$ 40%) and neutrally buoyant micro-particles^4^ and a thick coating regime was observed, which was attributed to non-Newtonian behavior of the mixture. In our work, three different particle volume fractions (20, 35 and 50%) were considered and we assumed no yield stress behavior in the pseudo suspension. The effective viscous regime is observed at the combination of high particle and binder volume fractions (compositions 02, 12, 22 and 21). At a higher solid loading, more particles will be near the boundary layer during extraction and entrained particles will create a rough surface topology. This newly generated transient roughness will help entrain more particles that are draining due to the capillary force, resulting in the multi-layer particle entrainment observed in the effective viscous regime.

### Influence of particle volume fraction

At low capillary number ($\le 3\times {10}^{-3})$, relatively smaller particles adhere in a heterogeneous pattern on the substrate, which can be attributed to the low polymer layer thickness demonstrated in Fig. 4. The experimental thickness at lower capillary number is higher than the theoretical thickness (${h}_{LP}$) due to the presence of large particles (avg. dia. 5.69 µm) and their polydisperse size distribution (Fig. 8). At 20% particle volume fraction (compositions 00, 10, and 20), the dipping mixture behaves similar to semi-dilute suspension, and lower particle coverage and coating thickness are observed compared to other compositions. In this regime, the particles mostly adhered as a single layer and assembled in a disordered cluster pattern. This behavior is likely caused by the drying polymer film that may pull the particles together to form the clusters. A transition zone can be observed as the particle volume increases to 35%, which also increases the capillary number. At capillary number > $3\times {10}^{-3}$, the upward convective flux increases, and the viscous drag force starts to dominate the particle adhesion. As a result, more particles adhere to the substrate, and multi-layer particle coating starts to form. At this range, the coverage transitioned to the effective viscosity regime for almost full coverage (> 90%) and after few layers of particle adhesion, the entrainment reaches to steady-state due the surface asperities created by the entrained polydisperse particles. Thus, with the combined effect of roughness due to asperities and the hydrodynamics created by the stirring motion, the thickness became steady as the capillary number increases, which is shown in Fig. 8.

### Influence of density mismatching

Due to the density mismatch between the particles and the liquid carrier system, external stirring energy is provided to counter the effect of gravity. Because of the larger particle size (> 5 µm), the van der Waals forces between IPs are not enough for particles to adhere to each other^61^, and thus, they do not coagulate. With the just suspending stirring speed, the heterogeneous mixture turns into a pseudo suspension, making it dippable. No study exists to predict the thickness or the particle coverage for a density mismatched heterogeneous mixture (negatively buoyant and polydisperse micro particles). In our study, we accounted for the density difference in both $h {\mathrm{and}} Ca$ by considering weighted volume fractions. While comparing the particle transfer matrices of the ‘pseudo suspension’ with the existing analytical expression of density matching suspension, the trends are similar. Thus, providing the stirring energy in the form of ‘just suspending speed’ into the heterogeneous mixture helped it to behave more like a ‘pseudo suspension’ and the density difference had little effect on the particle transfer as shown in Fig. 7. However, the effect of stirring energy or speed on such heterogeneous mixture has yet to be investigated fully.

### Influence of binder concentration

When the dipped substrate is withdrawn from the heterogeneous mixture, the solvent tends to evaporate from the substrate. During solvent evaporation, the concentration of polymer increases and the layer transitions from a viscous liquid to a gel-like solid which helps the particles to adhere to the coated substrate. The polymer coat around the particles acts as a deformable softshell on the hard solid particles, which increases the contact surface between particle and rod at the meniscus. Simultaneously, the increase in viscosity due to solvent evaporation and contact area increases the adhesion force acting on the particles. With an increase in binder volume fraction, the evaporated dense boundary layer will contain more binder molecules which increases the viscosity and decreases the drying rate. This will facilitate the entrainment of larger particles, increasing the particle coverage on the substrate and preventing draining of them from the substrate. The minimum film thickness required for neutrally buoyant particle entrainment ($${h}^{*})$$ on a plate geometry has been reported as $${h}^{*}\ge 2a$$ ^70^. Recent experimental studies suggested $${h}^{*}\ge 0.33a$$ for plate geometry^40^ and $${h}^{*}\ge 1.1a$$ for fiber geometry^55^. However, we found entrained particles at lower $${h}^{*}$$ which is further investigated in the next section. The transition between heterogeneous and effective viscous regime is observed in compositions 01, 11, and 21 where binder concentration varies with a fixed particle volume fraction (35%). Over 90% coverage is observed at 35% particle volume and 10.5% binder volume in the effective viscous regime. Thus, higher surface coverage can be achieved with high binder concentration at a fixed particle volume fraction.

### Influence of the withdrawal velocity

**Figure 9:**
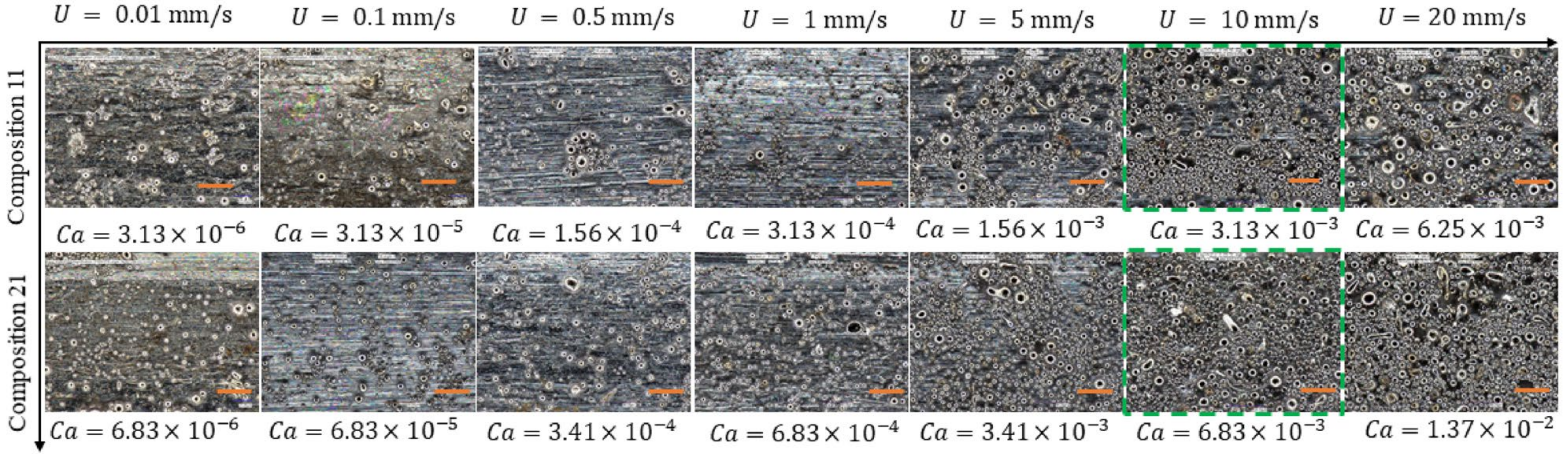
Surface morphology of the coated substrate at different withdrawal velocities at compositions 11 and 21. The picture was taken 1000X zoom. The scale bar is 50 µm. The green outlined images present the particle entrainment on optimized velocity.

The transition between the heterogeneous regime and the effective viscous regime is attributed to the capillary number, which is controlled with particle and binder volume fractions. However, withdrawal velocity, $U$ effectively changes the capillary number and the entrainment of particles. The convective flux on particles occurs due to the influence of solvent evaporation and capillary rise during the withdrawal process^51,79,80^. At a draining regime with a withdrawal speed (1 ~ 10 mm/s), the substrate moves faster than solvent evaporation^8^. However, at a lower withdrawal speed (< 1 mm/s), the convective flux is stopped by solvent evaporation since the influx of particles is limited here. Furthermore, with higher withdrawal velocity (> 10 mm/s), the spatial discrepancy in particle influx occurs due to the low solvent evaporation and gravitational effects, which resulted in a disordered cluster pattern, as shown in Fig. 9.

**Figure 10:**
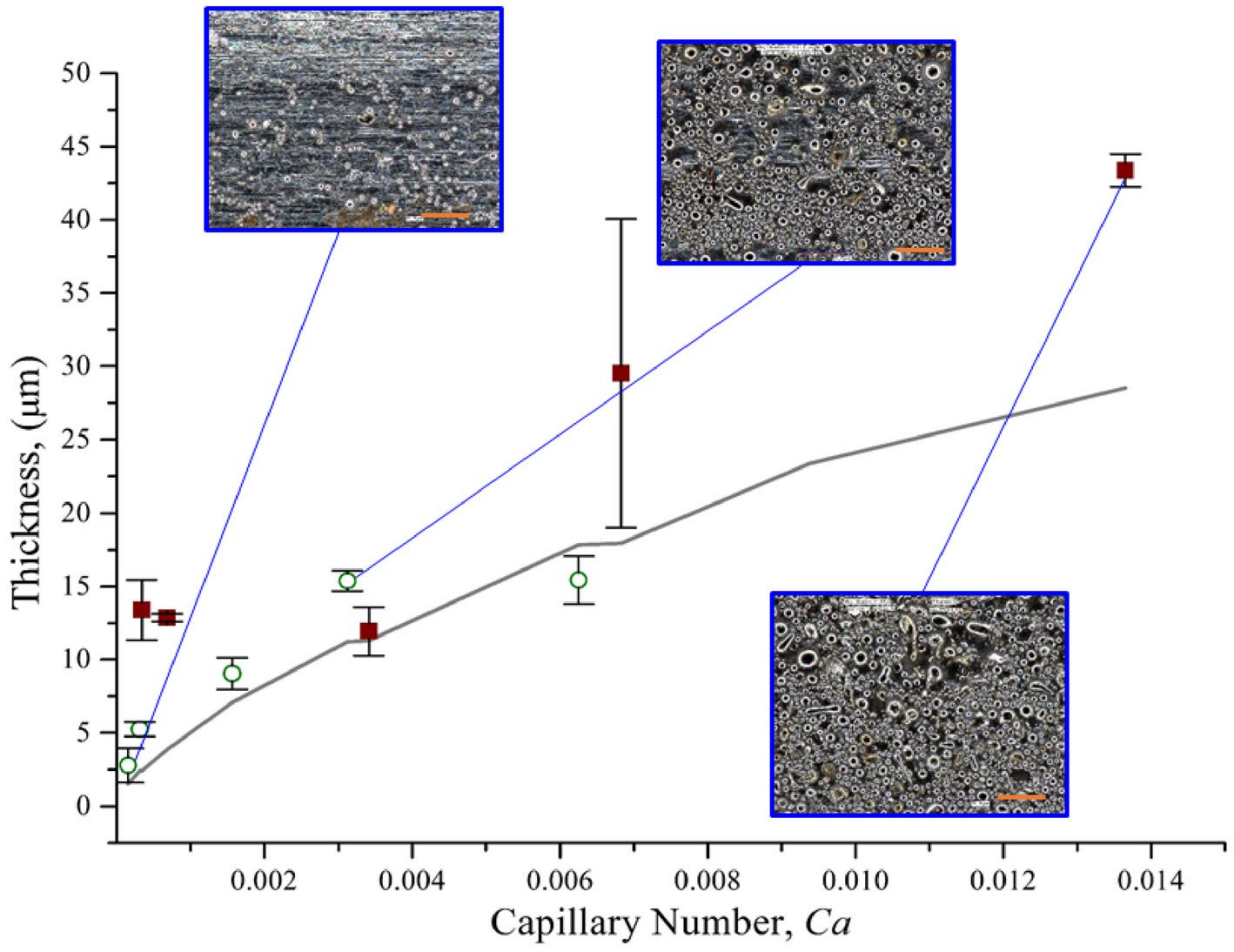
Thickness as a function of capillary number for different withdrawal velocities (0.5 to 20 mm/s). The grey line corresponds to the LLD law thickness. The hollow circle is for composition 11 (6.5% binder and 35% powder) and the solid square is for composition 21 (10.5% binder and 35% powder). The scale bar is 50 µm.

To investigate the critical film thickness, $${h}^{*}$$, for density mismatching system with stirring motion, the entrainment behavior was studied for two compositions (composition 11 and 21) with lower withdrawal velocity, as shown in Figs. 9 and 10. Below 5 mm/s, low particle coverage is observed and only small size particles are entrained which suggests a lower $$h$$. The film thickness $$h$$ is predicted with the modified power-law given by Eq. (1). For both compositions, particles can be seen entrained following the two-regimes discussed earlier. We did not observe a liquid regime or ‘zero-particle’ regime. There are two possible reasons for this observation: (i) particles are entrained at a lower $${h}^{*}$$, i.e., $$\alpha <1.1$$ or (ii) smaller particles ($$2a<5.6\,\upmu{\mathrm{m}})$$ from the poly-disperse distribution are entrained on the substrate, which will reduce the threshold capillary number ($${Ca}^{{*}})$$.

The average particle diameter for 0.01 mm/s was measured as 4.3 µm and 4.2 µm, which is smaller than the bulk particle average. For these particles, the threshold capillary number ${Ca}^{\boldsymbol{*}}$ for particle entrainment was calculated using Eq. (3) to be $5.81\times {10}^{-5}$ and $6.07\times {10}^{-5}$, respectively. For ${Ca}^{\boldsymbol{*}}$ below these values, no particles should entrain on the substrate. However, the capillary number for compositions 11 and 21 are as $3.1\times {10}^{-6}$ and $6.8\times {10}^{-6}$ at 0.01 mm/s speed, which are below ${Ca}^{\boldsymbol{*}}$. Additionally, in both circumstances, particles entrained for $\alpha =0.16$ and $\alpha =0.26$, respectively, which suggests that the critical film thickness, ${h}^{*}$, is significantly smaller than previously reported for particle entrainment. Since we have used polymer binder in our mixture, it facilitates the particle entrainment with lower liquid film thickness. A polymer rich boundary layer with higher viscosity is the possible reason for the particle to be entrained with lower $\alpha$ and hence ${h}^{*}.$

## Conclusions

In this paper, the entrainment of solid micro-particles was investigated on a cylindrical substrate withdrawn from a bath of pseudo suspension of non-Brownian particles dispersed in a Newtonian fluid with an evaporating solvent at 20% to 50% solid concentration. For the LCS, we found lower experimental thickness from the expected film thickness equation (Eq. 1) due to solvent evaporation. However, the addition of inorganic micro-particles modified the film thickness significantly.

After changing the volume fraction of the mixture (i.e., binder, particle) we observed two coating regimes. In semi-dilute suspension of 20% particle concentration, we observed the liquid film with a few clustered particles in an irregular pattern. This regime was observed in a low capillary number below $3\times {10}^{-3}.$ At a capillary number over $4\times {10}^{-3}$ more particles were entrained on the substrate at a uniform arrangement with 35% and 50% particle concentration. High binder volume fraction also facilitates particle adhesion in this regime. To explore a possible zero-particle regime, we changed the withdrawal velocity and observed particle entrainment below the threshold capillary number. Particle entrainment was observed with low liquid film thickness due to the evaporation of the solvent and generating a polymer rich dense layer at the interface to create a boundary layer that acts like a softshell for particle adhesion. The results also showed that smaller particles adhered on the substrate from the polydisperse distribution. From our analysis, we rationalized our experimental results with the theoretical predictions. Our results also provide a better understanding of the film thickness behavior from heterogeneous mixture preparation. Additionally, we found that with just suspending speed stirring energy, the density mismatched mixture behaves like pseudo suspension. The transferred particle matrices closely follow the analytical expression of a density matching suspension which demonstrate that the density mismatch effect can be neutralized with the stirring energy. This micro-particle transfer mechanism from density mismatched multi-phase mixture can be leveraged in coating processes for surface protection, surface modification, surface cavity filling, particle sorting and dust mitigation applications.

## Supplementary Information

Supplementary Information.

## Data Availability

All data generated or analyzed during this study are included in this published article as supplementary information files.
